# Virtual Reality Cognitive Therapy in Inpatient Psychiatric Wards: Protocol for a Qualitative Investigation of Staff and Patient Views Across Multiple National Health Service Sites

**DOI:** 10.2196/20300

**Published:** 2020-08-20

**Authors:** Poppy Brown, Felicity Waite, Sinéad Lambe, Laina Rosebrock, Daniel Freeman

**Affiliations:** 1 Department of Psychiatry University of Oxford Oxford United Kingdom; 2 Oxford Health NHS Foundation Trust Oxford United Kingdom

**Keywords:** virtual reality, therapy, inpatient psychiatric care, implementation

## Abstract

**Background:**

Patients in psychiatric wards typically have very limited access to individual psychological therapy. Inpatients often have significant time available, and an important transition back to everyday life to prepare for—but historically, there have been few trained therapists available on wards for the delivery of evidence-based therapy. Automated virtual reality (VR) therapy may be one route to increase the provision of powerful psychological treatments in psychiatric hospitals. The gameChange automated VR cognitive therapy is targeted at helping patients overcome anxious avoidance and re-engage in everyday situations (such as walking down the street, taking a bus, or going to a shop). This treatment target may fit well for many patients preparing for discharge. However, little is known about how VR therapy may be viewed in this setting.

**Objective:**

The objectives of the study are to explore psychiatric hospital staff and patients’ initial expectations of VR therapy, to gather patient and staff views of an automated VR cognitive therapy (gameChange) after briefly experiencing it, and to identify potential differences across National Health Service (NHS) mental health trusts for implementation. Guided by an implementation framework, the knowledge gained from this study will be used to assess the feasibility of VR treatment adoption into psychiatric hospitals.

**Methods:**

Focus groups will be conducted with NHS staff and patients in acute psychiatric wards at 5 NHS mental health trusts across England. Staff and patients will be interviewed in separate groups. Individual interviews will also be conducted when preferred by a participant. Within each of the 5 trusts, 1 to 2 wards will be visited. A total of 8-15 staff and patients per ward will be recruited, with a minimum total of 50 staff and patients recruited across all sites. Focus group questions have been derived from the nonadoption, abandonment, and challenges to the scale-up, spread, and sustainability (NASSS) framework. Focus groups will discuss expectations of VR therapy before participants are given the opportunity to briefly try the gameChange VR therapy. Questions will then focus on opinions about the therapy and investigate feasibility of adoption, with particular consideration given to site specific issues. A thematic analysis will be conducted.

**Results:**

As of May 15, 2020, 1 patient focus group has been conducted.

**Conclusions:**

The study will provide unique insight from patients and staff into the potential for implementing automated VR therapy in psychiatric wards. Perspectives will be captured both on the use of immersive technology hardware and therapy-specific issues in such settings.

**International Registered Report Identifier (IRRID):**

DERR1-10.2196/20300

## Introduction

### The Implications of Digital Technologies to Mental Health

There is a clear rise in the use of digital technologies, especially online apps, to deliver mental health treatments [1]. A second wave of digital treatments that use virtual reality (VR), increasingly being tested and shown to be effective in clinical trials, are likely to be implemented in services in the future [2]. VR therapy may be a particularly valuable tool in psychiatric wards. VR provides a safe and controlled setting for patients to practice entering, and coping with, challenging situations they may face at discharge. Therefore, it is important to assess the feasibility of implementing VR therapy in inpatient ward settings, and identify likely barriers and facilitators.

### Psychiatric Wards

Over the past 60 years, there has been an increasing move away from inpatient care toward the provision of care in the community whenever possible [3]. However, inpatient admission remains an important part of the care pathway when a person’s illness cannot be sufficiently managed in the community [4]. Qualitative investigations suggest that inpatient admission is needed to provide safety and protection from difficult environments, with many patients coming from places that they found to be too stressful and where they felt at risk of hurting themselves or others [5].

The shift in strategy toward community care has led to a reduction in the provision of inpatient beds. Bed numbers in England fell by 62% between 1987 and 2010, from almost 70,000 to fewer than 35,000 [4]. For adults in England, there are now just 18,000 beds, despite increases in the number of people in contact with mental health services [6]. The number of admissions to psychiatric wards has fallen accordingly, with a 19% reduction since 2012. However, bed occupancy remains high, at 95% in 2019 [6]. Average length of stay and numbers of involuntary admissions (ie, individuals detained under the mental health act) are also increasing [7]. Currently in the UK, the average length of stay in psychiatric wards is approximately 46 days. First admissions tend to be briefer, with an average length of 35 days. Length of stay is longer, with an average of 60 days, for those admitted involuntarily, compared to 37 days for those voluntarily admitted [8]. In 2019, 40% of admissions were involuntary, and the majority (62%) of all occupied bed days were by patients with psychosis [6], with these individuals also being the most likely to be detained [9]. It is clear that the need for inpatient admission remains, but with reduced capacity, the severity of illness required for admission has increased.

Inpatient wards are the most expensive form of care, with each acute adult bed costing up to GBP £180,000 (US $236,277.84) per year, the equivalent cost of supporting 44 people through a community mental health team over a year [10]. The lack of available beds and pressures to meet targets for lower bed occupancy rates [11] means ward staff are often forced to focus on achieving acute symptom reduction in patients rather than improvement in social functioning or coping ability [8]. Pressures are compounded by the limited availability of trained staff [12], a reliance on agency staff, and high levels of staff burnout [13]. Therefore, opportunities for staff-patient engagement in therapeutic relationships and collaborative care focused on recovery are limited [9,14]. Delivery of one-to-one or group psychological therapy is infrequent [15], with wards having very limited input from qualified psychologists [16] and treatment being predominantly pharmacological [12].

A further challenge to recovery is the lack of meaningful activities on wards, with patients often feeling bored and lonely [5]. Qualitative reports suggest that time is filled primarily with meals, smoking, and trying to look for someone to talk to [17], and that for some patients, the feeling of constant waiting is stressful and overwhelming [18]. One patient from a qualitative study described, “All you did was just sitting around, and there was nothing for you to do…no program to keep you busy…it’s not good…I stagnate” [19]. Both staff and patients recognize that the provision of meaningful occupation is central to recovery and wellness [20,21], but pressures on staff time often prevent it.

The lack of both therapy provision and engagement in meaningful activities means that patients are often unprepared for discharge. Patients can access escorted (and eventually, unescorted) leave from the ward [22]; however, it is unclear how frequently this forms part of the therapeutic preparation for discharge, in which leave, for example, is used to practice coping with some of the difficult situations that may have led to a patient’s admission in the first place. Consequently, although symptoms may be reduced upon discharge, patients can be ill-equipped with the skills needed to continue their recovery.

Leaving hospital often leads to the re-emergence of the pre-existing stressors that contributed to admission [23,24]. This may explain why the risk of relapse and rehospitalization immediately postdischarge is high [25]. Rates of suicide among patients in their first 3 months after discharge are also high, estimated at 100 times the global suicide rate, with a particular risk in the first week after discharge [23]. Significant anxiety about leaving hospital, sometimes known as “discharge grief,” is common [17]. There is a clear need for greater focus on safe transition and discharge preparation. To accomplish this, it is argued that wards must shift from a predominant focus on observation and monitoring of patients for acute symptom reduction, to one of active encouragement of patients to engage in activities and their own care management [5,10].

### Virtual Reality Therapy

Immersive virtual reality (VR) technology may provide a way of facilitating preparation for discharge. Difficulties interacting with the social world lie at the heart of most mental health problems [2], and it is clear that patients on wards require greater support to re-enter the external social world, which they previously found challenging [5]. In VR, it is possible to enter computerized simulations of scenarios that an individual finds difficult, while practicing powerful psychological techniques. This enables individuals to change the way they think, react, and behave in such scenarios. Automating VR therapy means individuals can make use of the therapy even when there is a lack of highly trained staff. The potential for using VR in therapy has been well recognized over the past 25 years, but the development of consumer kits—and with it, the possibility of scaling VR therapy—has occurred only recently [2]. The hardware consists of a computer that generates an image, a display system that presents sensory information, and a tracker that feeds back the user’s position and orientation to update the image.

VR has several key advantages over traditional face-to-face therapy. Patients are more willing to enter VR simulations of the situations they find anxiety-provoking because they know the simulations are not real. At the same time, individuals respond the same in VR, psychologically, emotionally, and physiologically, as they do in corresponding real-world environments [26]. Therefore, any learning that has occurred in VR transfers to the real world [27]. Consequently, VR provides a way of immersing individuals in the very environments in which they require practice when they are too fearful or, as is the case in inpatient wards, unable to do so in the real world.

The gameChange VR therapy utilizes this very concept to treat anxious social withdrawal [28]. Many individuals with mental health disorders (particularly, serious mental disorders such as psychosis) withdraw from everyday social activities due to anxiety. Two-thirds of patients with schizophrenia have levels of anxious avoidance equivalent to agoraphobia [29]. The key mechanism utilized by the gameChange therapy concerns safety-seeking behaviors, also known as defenses. Defenses are behaviors that individuals employ to help them feel safer. However, these behaviors actually serve to maintain thoughts and feelings of fear by preventing the learning of disconfirmatory evidence. Dropping defense behaviors during difficult situations allows patients to relearn concepts of safety [30]. Therefore, the gameChange therapy identifies patients’ defenses and encourages them to try dropping their defenses in virtual social situations, thus helping to achieve new learning of feelings of safety and confidence. The current gameChange therapy includes 6 virtual scenarios: a street, café, pub, GP surgery, corner shop, and bus, with 5 levels of difficulty within each scenario. The user-centered design process for this therapy has been described in a recent paper [31].

Notably, the gameChange therapy is automated. A virtual coach, Nic, guides patients through each situation and suggests new behaviors to test out. Therefore, the therapy does not require a trained cognitive behavioral therapist to deliver it. While there is still someone in the room with the patient, this individual can be a peer supporter, psychology assistant, social worker, or health care assistant. This individual’s role is to set up the equipment and provide support and encouragement. As such, VR delivery staff require only brief initial training and then ongoing supervision with a psychologist. The gameChange therapy is currently being tested in a multi-site randomized controlled trial [28]. Within the trial, patients are offered 6-8 weekly therapy sessions supported by a member of staff, typically an assistant psychologist, peer support worker, or clinical psychologist. Sessions take place either in the participant’s home or local mental health base.

Many studies have shown the effectiveness of VR therapy for patients with a range of mental health problems [32-34]. Using these therapies on wards could provide a unique opportunity for helping patients prepare for discharge through the experiential practice of a range of everyday situations. The delivery of an automated VR therapy can be facilitated by a wider range of professionals on the ward and is not constrained to a therapist trained in one-to-one psychological therapies. Higher doses, perhaps daily, would be feasible.

If VR headsets were accessible on wards, additional, freely available VR programs such as physical activity games, relaxation, and meditation exercises could also be used by patients as therapeutic activities that lessen boredom and enhance recovery. The feasibility of this has increased greatly due to continuous hardware improvements and a reduction in costs. This means VR equipment now requires less space, is less technical, and is more user-friendly than it was previously.

### Implementation Framework

Implementation frameworks provide an overview of the factors that typically shape and influence the implementation process [35]. We used the nonadoption, abandonment, and challenges to the scale-up, spread, and sustainability (NASSS) framework for health care technologies [36] to inform the study’s design. The NASSS draws together a number of implementation models and theories, and covers 7 domains relating to health care technology implementation: the condition or illness, the technology, the value proposition, the adopter system, the organization, the wider context, and embedding and adaptation over time. Challenges regarding each domain are classified as simple (straightforward, predictable, few components), complicated (multiple interacting components or issues), or complex (dynamic, unpredictable, not easily disaggregated into constituent components). Staff and patients are in a position to inform 3 of these domains with regard to implementation of VR therapy: the condition and illness that the therapy is designed for, the intended adopters of VR therapy, and the organization. Other frameworks were also considered, such as the normalization process theory (NPT) [37]. However, the NASSS framework covers a wider range of potential barriers and facilitators to implementation that may be relevant at any point from design through to continued implementation, whereas NPT is more retrospective in nature.

### Objectives

The study objectives are threefold: (1) to obtain initial expectations of staff and patients about VR and VR psychological therapy; (2) to gain staff and patient views of an automated VR therapy (gameChange) after trying it; (3) and to identify potential differences and requirements for implementation across health care sites.

## Methods

To increase the methodological quality and reporting, the presentation of the study will follow the guidance of the 32-item consolidated criteria for reporting qualitative research (COREQ) [38].

### Ethical Review

The gameChange trial received Health Research Authority (HRA) approval and Health and Care Research Wales approval (IRAS 256895, The gameChange Trial). The trial received ethical approval from the NHS South Central - Oxford B Research Ethics Committee (19/SC/0075). The trial has been registered (ISRCTN17308399) and the protocol published [28]. The present study received ethical approval as part of a substantial amendment.

### Patient and Public Involvement

In line with the guidance for reporting involvement of patients and the public (short form; GRIPP2-SF [39]) we report the aims, methods, results, and reflections on patient and public involvement (PPI).

There has been considerable PPI in the development of the gameChange therapy and the running of the trial. Within this study, the aim is to ensure all study documentation (topic guide, information sheet, and consent form) is engaging and understandable, and to involve service users in the design of the study. PPI will also be used to discuss the analysis and interpretation of results. A lived experience advisory panel (LEAP), facilitated by the McPin Foundation, contributed to the development of the study. The LEAP comprises 10 individuals from across the 5 study sites. All study documentation was sent electronically to the LEAP for feedback, and an in-person discussion about the study design took place. An additional in-person session will take place to discuss the analysis and results. Many areas of the study documentation were rephrased to make them more inclusive and comprehensible, and many suggestions for how to maximize engagement in focus groups were given. These included key times on the ward to avoid (eg, visiting hours, meal and medication times), reducing the power dynamic in focus groups (eg, by emphasizing that the researchers are here to learn from participants, not the other way round), ensuring the researchers state that the focus group ground rules also apply to themselves, and asking certain questions without making people uncomfortable (eg, by offering post-it notes or asking a question before a break).

Therefore, PPI has been a helpful influence on the study. As the LEAP had been involved with the gameChange trial, they were familiar with the VR that would be demonstrated, and the LEAP was thus well placed to reflect on how this would work in the focus groups. Several members had also been inpatients themselves, allowing them to give important advice about how focus groups could best be conducted on the wards.

PPI was considerable; however, involvement could also have been further strengthened. For example, not all 10 LEAP members were able to attend the in-person meeting. If time had allowed, another in-person meeting may have enabled the incorporation of a greater number of viewpoints.

### Context of Data Collection

There are likely to be a number of challenges affecting the data collection process. Wards can be chaotic environments, with unpredictable events and many patients experiencing high levels of distress, making the facilitation of focus groups difficult [16]. The staff pressures and shortages typically seen on wards may mean it is difficult for staff to schedule time for a focus group or interview in advance. For those who are able to take part, time may be limited, preventing the discussion of all relevant topics. In addition, some wards may not always have a suitable room available for conducting focus groups and interviews, so the researchers expect time constraints for when they can conduct focus groups or interviews. This will be compounded by the need to avoid key times on the ward, such as during ward rounds, medication dispensary, visiting hours, meal times, and any structured activities offered on the ward. To minimize these issues, the researchers will aim to be as flexible as possible in their approach, but challenges and disruptions to data collection are nonetheless expected.

### Participants

Staff working in either the delivery or management of clinical care on the wards will be invited to take part in focus groups or individual interviews. National Health Service (NHS) patients staying on wards will be recruited according to the following inclusion criteria: (1) participants are willing and able to give informed consent for participation in the study; (2) participants are 18 years old or older; (3) participants are willing to consent to being audio-recorded; (4) participants have sufficient English language skills to participate in the focus group or interview. The exclusion criteria will include high levels of associated risk to self or others through participation in the study (eg, actively suicidal), and photosensitive epilepsy (for which use of VR is not recommended). Researchers will assess a participant’s capacity to consent after the participant has read the information sheet and before they sign the consent form. Patients will receive a small payment for taking part.

### Sampling and Recruitment

The gameChange trial is recruiting from 5 NHS mental health trusts across the UK: Avon and Wiltshire Mental Health Partnership NHS Trust, Greater Manchester Mental Health NHS Foundation Trust, Cumbria Northumberland Tyne and Wear NHS Foundation Trust, Nottinghamshire Healthcare NHS Foundation Trust, and Oxford Health NHS Foundation Trust. Principal investigators (PIs) and trial coordinators will be at each site. The trial is open to patients from all mental health services, but to date, almost all participants are outpatients. We will work with the PIs and trial coordinators to approach leads of psychiatric wards at each site. Only acute psychiatric wards will be visited rather than rehabilitation wards, given these are the most numerous type. We aim to visit an equal number of male and female wards.

We aim to visit 1-2 wards within each of the 5 trusts, and include 8-15 total participants (staff and patients) from each ward. A minimum total of 50 staff and patients will be recruited across all sites. Due to the busy nature of wards and frequent lack of room availability, convenience (volunteer) sampling will be used in the first instance. Purposive sampling will then be used to ensure that a range of staff are seen (ie, those who are involved in decision-making as well as those who are more directly involved in day-to-day clinical care).

### Procedure

In the weeks leading up to the site visit, staff and patients will be informed of the study and focus group dates will be arranged. Staff and patients will receive participant information sheets and be given time to discuss this with others. The researchers will predominantly rely on members of ward staff to initially introduce the study and go through the information sheet with patients, given staff will be more familiar to patients. Before taking consent, the researchers will be available to take participants through the information sheet again and answer any questions. After consenting, a demographic questionnaire will ask participants their age, gender, and ethnicity. Staff will also be asked about their job roles. Patient diagnosis will not be recorded, given that patients themselves may not be willing or able to disclose this, and we do not wish to add to staff burden by asking them to provide this patient information. The first author (PB) will lead all focus groups. There will be a cofacilitator that is likely to vary by site. A member of staff from the ward may also be present during patient focus groups and interviews. Each of the wards will be visited multiple times to ensure participation is open to as many different patients and members of staff as possible. All data collection will take place on the ward.

Focus groups and interviews will initially ask questions relating to the study’s first objective (to obtain the initial expectations of staff and patients about VR and VR psychological therapy) before giving all participants the opportunity to put on a VR headset and try the therapy for a few minutes. They will meet the coach, Nic, and try out 1 level of 1 scenario. Participants will choose which scenario and level they enter, although patients will be encouraged to only try easier levels. Participants will also be observed while they try the VR therapy, and potentially videotaped if they give permission. Observations will be recorded in the researchers’ field notes. Further questions will then focus on objectives 2 (to gain staff and patient views of the gameChange automated VR therapy after trying it) and 3 (to identify potential differences and requirements for implementation across health care sites). If any participants leave the focus groups before the end, we do not plan to collect data on the reasons for withdrawal. This is for two reasons: firstly, it is expected to be practically difficult to follow up with a participant who leaves; secondly, participants are told that they may withdraw from the focus groups at any point without the provision of a reason, so as not to make anyone feel obliged to stay. Any data that they have provided prior to leaving will be included in the analysis.

### Focus Groups and Interviews

Focus groups were chosen as the primary mode of data collection because they allow individuals to consider ideas together while also highlighting differences in thoughts and ideas between participants [40]. They also allow participants to express ideas spontaneously, in a way that is less structured or influenced by the researchers’ prejudices [41]. Given most participants are expected to be unfamiliar with VR, a group setting is likely to be helpful for allowing individuals to consider a range of viewpoints and questions raised by other group members to inform their opinions. The group setting is also likely to be most constructive for generating ideas about potential challenges around the implementation of VR therapy, as well as solutions to challenges, because individuals can build upon each other’s suggestions. We aim for each focus group to contain 3-6 participants; however, this will vary depending on staff and patient availability. Wards are a challenging environment for such research, and pragmatism is needed. In particular, it is expected to be difficult to have multiple staff members available at the same time, so a number of single or joint interviews may be necessary. Individual interviews will also be conducted if a participant would prefer. For example, a number of patients might find a group setting difficult, and some members of staff may prefer to express their views privately. Focus groups are expected to last anywhere between 45 minutes to 2 hours. Individual interviews may be shorter. To limit the length of time staff are required to be available at any one time, the possibility of splitting the focus group or interview into 2 sessions will be offered.

### Topic Guide

Informed by the NASSS framework, the semistructured topic guide has been created to cover all 3 objectives. PB created a first draft of the topic guide, which was then revised following feedback from FW, DF, the LEAP, 2 experts in qualitative research, and a pilot with colleagues. The topic guide will be reviewed after conducting the first focus groups, and then again at a later stage of data collection and analysis. Changes may be made in response to participant feedback (eg, if focus groups are too long for participants, or if it becomes clear that a certain topic is being under or overexplored). Significant changes to the topic guide will be reported. A copy of the topic guide can be viewed in Multimedia Appendix 1.

### Analysis

Focus groups and interviews will be audio-recorded and transcribed verbatim. Field notes from each focus group or interview will also be transcribed. Field notes will record factors such as group dynamics and nonverbal cues to add context to the transcript of the audio recordings. For practical reasons, transcripts will not be returned to participants for comment or correction.

A thematic analysis will be conducted [42]. All data will be entered into NVivo (version 12.0, QSR) [43] in order to provide a transparent audit trail. PB will read and reread transcribed data to ensure familiarity before developing a preliminary coding framework. In line with recommendations [44], there will be team reviews of the coding framework, regular team consultation, and multiple coding for a number of interviews. Details regarding each code will be recorded in memos in Nvivo. Themes will be derived from the data. Data saturation will be discussed as the study progresses. Diverse cases and minor themes will be presented, as we consider breadth as important as frequency. A meeting with the LEAP will be set up in order to discuss the thematic analysis and consider interpretations of the results.

### Reflexivity

Researchers conducting the focus groups and analyzing the results will consider how their own backgrounds may impact data collection and analysis. PB will keep a reflexive log. Details of the research team and reflexivity will be reported in the full manuscript in line with COREQ guidelines [38]. However, reflexivity has also been considered at an early stage, prior to starting recruitment to the study.

All the researchers who will be conducting focus groups have been involved in the design or use of VR therapy for psychosis. Thus, existing knowledge, expectations, and hopes regarding VR therapy may impact how the focus groups are conducted. A number of groups may be cofacilitated by a clinical psychologist, and others may be cofacilitated by an assistant psychologist, which may impact the data in terms of both the cofacilitators’ actions (eg, how questions are asked) and how participants respond to the different roles. To try to minimize these potential biases, PB and the cofacilitators will aim to stay close to the interview schedule, as this was created largely from the NASSS implementation framework, not just the experiences and expectations of the authors.

## Results

As of May 2020, data collection for 1 patient focus group with 3 participants has been conducted, and coding is underway.

## Discussion

### Prospects

This protocol describes the plan for a multi-site qualitative study with patients and staff, assessing the feasibility of implementing VR therapy in inpatient psychiatric wards. As part of this process, NHS staff and patients in psychiatric wards will be able to try out and provide their feedback on the gameChange automated VR therapy. The study will provide insight into the degree to which VR therapy might be suitable for inpatient wards, and identify barriers and facilitators to implementation. Studies making use of implementation science should aim to produce generalizable knowledge [45]. As such, this study can also be contextualized as an investigation of the potential implementation of digital psychological therapies more generally in psychiatric wards.

### Limitations

There are several limitations to the methodology used in the study. We will only be recruiting from acute psychiatric wards; therefore, results may not generalize to all types of wards (such as rehabilitation wards). Similarly, the wards that agree to take part may be those that are currently not experiencing significant staff shortages, which may also limit the generalizability of findings.

It has been suggested that participants in implementation studies may represent a more highly motivated group of service users who are less representative of the whole population [46]. This may be a limitation of the participant group we recruit. Patient diagnosis will also not be recorded, nor will patients be asked about their specific current experiences and difficulties. Therefore, we will not know what kinds of problems most patients are experiencing unless they discuss them in the groups. In addition, while a focus group environment has a number of benefits, a proportion of participants may not feel entirely comfortable in this setting. This could be due to low self-confidence, conflicts between individuals on the ward, or hierarchical staff roles. Consequently, a number of individuals may not fully share their views. It is hoped that offering individual interviews may help to mitigate this problem, but it is still likely to be present.

### Strengths

The study methodology also has several strengths. First, multiple stakeholder involvement is considered important for implementation research [45,47]. Thus, conducting focus groups with staff of varying professional groups and patients is a particular strength of the study; a wide selection of viewpoints is likely to be gained. Second, conducting the study at 5 NHS mental health trusts across the UK will help to increase the generalizability of the results, and allow comparison between different locations. Third, the study methodology and documentation has received feedback from our LEAP, helping to ensure the study will be engaging and acceptable to patients. Fourth, the gameChange VR therapy has been designed to help with the very problem that many patients on wards are struggling with: coping with everyday environments. Therefore, it is likely to fit well with the goals of both staff and patients on wards. Finally, the majority of implementation research is retrospective [47]. This study benefits from prospectively assessing feasibility of implementation in this setting. Prospective assessment of digital interventions allows for optimization prior to implementation, in order to ensure long-term use and the meeting of clinical and scientific standards [48].

It is important to consider how health care technologies can be integrated into existing health services [49]. There have been significant recent advances in digital mental health care. This study will provide valuable insight into how one particular emerging health care technology, VR, might fare in implementation in psychiatric inpatient wards.

## Appendix

Multimedia Appendix 1 Focus group topic guides.

## Abbreviations

GRIPP2-SF: guidance for reporting involvement of patients and the public - short form
LEAP: lived experience advisory panel
NASSS: nonadoption, abandonment, and challenges to scale-up, spread, and sustainability
NPT: normalization process theory
PI: principal investigator
PPI: patient and public involvement
VR: virtual reality
