# Supplementary material for: Virtual Reality Cognitive Therapy in Inpatient Psychiatric Wards: Protocol for a Qualitative Investigation of Staff and Patient Views Across Multiple National Health Service Sites
Source: JMIR Res Protoc. 2020 Aug 20;9(8):e20300. doi: 10.2196/20300 (PMC7471884; doi:10.2196/20300)
Supplement: Multimedia Appendix 1 [file resprot_v9i8e20300_app1.docx]

**Topic Guides**

**Staff topic guide**

1. Has anyone ever tried Virtual Reality?
   1. What do you know about it? How did it make you feel?
2. What are your views on having psychological therapy of any sort available to patients on wards?
3. How do you think patients would feel about wearing a headset like this?

*Explanation of VR therapy*

1. What are your initial thoughts about this?
2. Is this relevant to anything you do with patients?
3. Can you describe any immediate or initial concerns you have about VR therapy, if any?

*Demo of VR therapy*

1. Do you think this VR therapy could help patients, and if so, in what ways?
2. The technology will keep improving so that things look increasingly realistic and the characters may become more interactive. Apart from these sorts of things, is there anything about the therapy you would change if it were to be implemented on the ward?
3. What are some of the things that surprised you about it?
   1. What did you think of Nic, and how it is automated?
4. How would your patients feel using the VR?
5. What are the characteristics of your patients that might affect whether or not they benefit from VR therapy?
   1. Can you give an example of someone who would enjoy it?
   2. And someone who would have trouble about it?
   3. What’s different about these patients?
6. How might having VR on the ward work?
   1. Who could you see delivering therapy to patients? Why or why not might these people be willing to learn to deliver it? Might this change over time?
   2. How often could you see patients using it and when? And where?
   3. How does it fit with the current delivery of treatment on the ward?
7. What concerns do you have VR therapy impacting on patient safety or disrupting care?
8. In what ways might VR therapy make care more time-consuming?
   1. And in what ways might it save time?
9. In what ways might having VR on the ward affect the role of staff?
   1. In what ways might it make someone’s job more difficult? Or easier?
10. What training/supervision would be needed in order to feel confident delivering the therapy to patients?
11. In what way does the therapy fit with or get in the way of any service goals?
12. What do you feel would need to happen for VR therapy to work in practise?
    1. What things would make it easier?
    2. What would make it difficult?
13. What previous examples are there of something new/a change being introduced into the ward?
14. What things about your service might make it either particular easy or hard, or more or less relevant, to have VR therapy in practise?
    1. Physical location e.g. city centre
    2. Typical patient group
    3. Staffing
    4. Ward logistics
15. Do you have any other comments, recommendations or concerns?

**Patient topic guide**

1. Has anyone ever tried Virtual Reality?
   1. What do you know about it? How did it make you feel?
2. What are your views on having psychological therapy of any sort available to patients on wards?
3. What are you first thoughts about wearing a headset like this?

*Explanation of VR therapy*

1. What are your first thoughts on this therapy?
2. Do you have any immediate or initial concerns about VR therapy?

*Demo of VR therapy*

1. What did you think of the VR therapy?
2. How would you feel about using VR therapy while on the ward?
   1. Would you use it if it were available? Why or Why not?
   2. What do you like about it?
   3. What do you dislike about it?
   4. In what way is it helpful?
   5. What surprised you about it?
   6. How easy did you find the VR to use?
3. How did you feel about the character (Nic) guiding you and not a real life therapist?
4. What concerns do you have about the therapy or about VR?
5. The technology will keep improving so that things look increasingly realistic and the characters may become more interactive. Apart from these sorts of things, what might you change about the therapy if you could change anything?
6. What about your experiences that brought you to the ward might affect your use of VR?
7. Who would you like to deliver VR therapy to you?
8. What availability/access would you want to it?
9. What problems do you think there would be having the therapy available on the ward?
10. Do you have any other comments, recommendations or concerns?
